# Anti-tumor action of trichosanthin, a type 1 ribosome-inactivating protein, employed in traditional Chinese medicine: a mini review

**DOI:** 10.1007/s00280-013-2096-y

**Published:** 2013-02-03

**Authors:** Ou Sha, Junfei Niu, Tzi-Bun Ng, Eric Yu-Pang Cho, Xiaoyuan Fu, Wenqi Jiang

**Affiliations:** 1 School of Medicine, Shenzhen University, Shenzhen, China; 2 Faculty of Medicine, School of Biomedical Sciences, The Chinese University of Hong Kong, Hong Kong, China

**Keywords:** Trichosanthin (TCS), Ribosome-inactivating protein, Anti-tumor, Apoptosis

## Abstract

Trichosanthin (TCS) as a midterm abortifacient medicine has been used clinically in traditional Chinese medicine for centuries. Additionally, TCS manifests a host of pharmacological properties, for instance, anti-HIV and anti-tumor activities. TCS has been reported to inhibit cell growth of a diversity of cancers, including cervical cancer, choriocarcinoma, and leukemia/lymphoma, etc. This article purported to review the various anti-tumor activities of TCS and the mechanism of apoptosis it induced in these tumor cells. These research progresses provide an insight into cancer research and treatment as well as disclose new pharmacological properties of the ancient but popular Chinese medicine.

## Introduction

Trichosanthin (TCS) is a renowned traditional Chinese medicine and still used in clinics in China for inducing midterm abortion. TCS belongs to the family of plant proteins known as ribosome-inactivating proteins (RIPs), which can attack the ribosomes of eukaryotic cells by virtue of their rRNA N-glycosidase activities [1–3]. This results in the inhibition of protein synthesis and ultimately the death of eukaryotic cells. RIPs are classified into two major types [4–6]. Type I RIPs consist of only a single rRNA-cleaving domain. Most of them are constructed of a single intact polypeptide with a molecular weight around 30 kD. Type II RIPs comprise one or more rRNA cleaving, amino-terminal domain(s), which are commonly called A chain(s), and resemble type I RIPs. They are each linked by a disulfide bond to a structurally unrelated carboxyl-terminal domain called B chain [2].

TCS which possesses only one polypeptide chain is a classic example of a type I RIP. As TCS is devoid of a cell-binding B chain, it has less cytotoxic effects than most of type II RIPs, such as ricin, and more selective toxicity to cultured tumor cells, ovulated oocytes, HIV-infected macrophages, and normal human macrophages. [7, 8]. Recently, more and more studies were focused on its anti-tumor and apoptotic activities. The aim of this article was to review the recent progress in research on the anti-tumor activity of TCS.

## Introduction to TCS

Trichosanthin (TCS), or Tin Hua Fen, is obtained from the root tuber of *Trichosanthes kirilowii* Maxim belonging to the Cucurbitaceae family. It has been employed for centuries in traditional Chinese medicine as an abortifacient drug in early and mid-gestation. Only in recent decades were its enzymatic activities recognized as characteristic of type I RIPs [9]. Although TCS has been adopted for abortion purposes for hundreds of years in China, scientific investigations on TCS commenced in around 1966 and many of the early studies were not published [10]. The first publication on TCS appeared in 1976 [11]. Since then, more and more biochemical and pharmacological investigations have been conducted by researchers in Hong Kong, China, and Western countries [6, 12].

TCS is composed of a single polypeptide chain with a molecular weight of 27 kDa. Its amino acid sequence demonstrates homology to the A chains of many type II RIPs, such as ricin A chain [13–15]. It inhibits protein synthesis through cleavage of the N–C glycosidic bond of adenine 4324, the 4324th base of 28S rRNA. This action renders the ribosome incapable of binding elongation factor 2 and therefore terminates translation [16, 17].

TCS owns a wide spectrum of biological and pharmacological activities (Table 1). It produces adverse effects on reproduction in the mouse by causing follicular atresia and degeneration of ovulated oocytes [7]. In fertilized animals, TCS elicits death of syncytiotrophoblasts of placental villi, and consequently, the embryo fails to develop [10, 11]. This action has been related to its uptake by placental trophoblast cells. TCS also exhibits immunomodulatory (immunosuppressive), anti-tumor, anti-viral, and anti-human immunodeficiency virus (HIV) activities [6, 16]. Its anti-HIV activity is attributed to inhibition of the replication of HIV and cytotoxicity to HIV-infected macrophages and lymphocytes [8, 18]. The fact that TCS is applied in the treatment of choriocarcinoma is consistent with its abortive activity, since this tumor also originates from fetal trophoblast cells [13]. The anti-tumor activity of TCS is the main focus of this article.

**Table 1:** Summary of various pharmacological activities of trichosanthin including anti-cancer activity

Pharmacological activity	Ref. no.
Induction of atresia of ovarian follicles and inhibition of steroidogenesis in gonadotrophin-primed immature mice	[7]
Induction of abortion	[10, 11]
Anti-HIV and anti-HIV enzyme	[16, 18]
Neurotoxicity	[5]
Immunomodulatory	[6]
Inhibition of protein synthesis	[6]
RNA N-glycosidase activity	[6]

## Anti-tumor activity of TCS

### Effects of TCS on choriocarcinoma

TCS is well known to exert deleterious effects on reproduction and is a clinical medicine for abortion. In the mouse, it engenders follicular atresia and degeneration of ovulated oocytes [7]. In fertilized animals, TCS causes necrosis of the syncytiotrophoblasts of placental villi, and consequently, the embryo fails to develop [19–21]. Therefore, the anti-tumor effects of TCS were first tested on the cells of choriocarcinoma, a malignant trophoblastic cancer, usually of the placenta (Table 2).

**Table 2:** Summary of TCS anti-tumor activities, including in vivo and in vitro experiments

System	Tumor	Cell line (in vitro)	Animal (in vivo)	Ref. no.
Female reproductive	Choriocarcinoma	JAR & BeWo		[22–28]
	Cervical cancer	HeLa & Caski		[30–41]
			Kunming mouse (U14-cells)	[42]
	Breast cancer	MDA-MB-231 & MCF-7	Nude mouse (transplanted)	[55]
Blood	Leukemia & lymphoma	K562, Jurkat, HUT78, Mo l-t 4, Jurkat, CEM, Raji & Daudi		[8, 37, 40, 43–45]
Digestive	Hepatoma	H35, HepA-H & HepG2		[12, 23, 30, 31]
	Colon carcinoma	LoVo & CT-26	By Sw-1116 cells	[49–51]
	Stomach adenocarcinoma	MCG803		[52]
Respiratory	Lung cancer		Nude mouse (A549 cells)	[53]
			Lewis rat	[54]
Male reproductive	Prostatic cancer	RM-1		[56]
Other	Melanoma	B16		[57]

About two decades ago, Dai and colleagues conjugated colloidal gold to TCS molecules and found that TCS specifically entered cultured trophoblast and choriocarcinoma JAR cells via receptor-mediated endocytosis [22]. The finding was subsequently corroborated by other investigators [23, 24]. The LDL receptor-related protein-1 (LRP1) has been suggested as a major receptor for phagocytosis of TCS in cultured JAR and BeWo cells, which might be the molecular basis of the abortifacient and anti-choriocarcinoma activities of TCS [25, 26]. Influx of calcium and production of reactive oxygen species (ROS) were also observed in TCS-treated JAR cells, and ROS production might be a consequence of calcium ion signaling [27–29].

### Effects of TCS on cervical cancer

When TCS was added to cultured tumor cells, it brought about a reduction in the uptake of radioactive precursors for protein synthesis [30]. This suggested that TCS killed cells by virtue of its RIP activity. In addition, the anti-tumor effect of TCS on cervical cancer has also been revealed in many studies. Most of them are based on in vitro experiments, with only a few based on observations in vivo. Scientists first tested the toxic effects of TCS on HeLa cervical cancer cells. TCS manifested significant inhibitory effects [31, 32]. It heightened cytosolic calcium and suppressed intracellular cAMP/protein kinase C (PKC) levels via PKC inhibition [33–36]. Different from usual RIP activities, TCS brings about apoptotic cell death in HeLa cells, and activation of caspases 8, 9, and 3 has been observed [37]. The upregulation of ER chaperone immunoglobulin heavy chain-binding protein (BiP) and C/EBP-homologous protein (CHOP) and activation of caspase 4 suggest the participation of the endoplasmic reticulum stress pathway in TCS-induced HeLa cell apoptosis [38]. Recent studies have also demonstrated the toxicity of TCS on cervical cancer CaSki cells, and that TCS plays a role in demethylation by inhibiting DNA (cytosine-5)-methyltransferase 1 (DNMT1) enzyme activity and DNM1 mRNA and protein expression in CaSki cells [39, 40]. The demethylation of TCS in HeLa cells takes place via attacking TSLCl and p16 genes [41, 42].

Only one article has reported the effect of TCS on cervical cancer in vivo. Zhang et al. [43] established an animal model of cervical cancer by repeated injections of mouse U14 cell line into Kunming mice. TCS potentiated the humoral immunity in mice at a dose of 0.2 mg/kg.

### Effects of TCS on leukemia/lymphoma

Another cancer that has been reported to be sensitive to TCS is leukemia/lymphoma. As early as in 1990s, TCS was found to be toxic to leukemia/lymphoma cells in vitro [8, 44]. A decade later, TCS was reported to exert an anti-tumor action on chronic myelogenous leukemia K562 cells and acute T cell leukemia Jurkat cells [38, 42, 45]. TCS caused a down-regulation of p210Bcr-abl and its downstream signals, resulting in tyrosine kinase inhibition in K562 cells [46]. Both PKC inhibition and caspase 3 activation are involved in TCS-induced apoptosis in K562 cells [45]. Under similar conditions, TCS is more toxic to HUT78, MOLT-4, Jurkat, and CEM cells originating from T lymphocytes and macrophages than Raji and Daudi cells from B lymphoma [42].

### Effects of TCS on other cancer cells

TCS has been reported to be effective against a variety of other tumors, including hepatoma, colon carcinoma, stomach cancer, lung cancer, breast cancer, prostate cancer, and melanoma. Some early research reported that TCS did not exert much toxicity to hepatoma cell lines, including H35 and HepA-H [23, 31, 32]. However, when epidermal growth factor (EGF) is conjugated to TCS, the immunotoxin EGF-TCS is toxic to hepatoma cells, for example, BEL-7402, MCF-7, and BGC-823 [47]. In addition, EGF-TCS also has in vivo anti-hepatoma effects when the hepatoma animal model is constructed by injection of BEL-7402 cells [48]. Actually, a TCS-based immunotoxin or TCS conjugated with a specific cell binding molecule has also been intensively studied. However, it is not the main focus of this minireview [49].

Furthermore, dexamethasone enhances the effects of TCS on apoptosis in HepG2 cells by inhibiting the NF-κB signaling pathway, which highlights the possibility of combined drug application of TCS and dexamethasone in the clinical treatment of hepatoma [12]. Studies also disclose that TCS exhibits an anti-colon carcinoma effect in both in vitro and in vivo experiments. When TCS gene is cloned and expressed in colorectal carcinoma LoVo cells, TCS evokes apoptosis in these cells [50]. Additionally, not only colon carcinoma cell line CT-26, but also in vivo colon carcinoma produced by Sw-1116 cells, is sensitive to TCS toxicity [51, 52]. Furthermore, TCS produces toxic effects on MCG803 cells of the stomach adenocarcinoma, another cancer of the digestive system [53].

An animal model of lung caner was created by administration of A549 cells to nude mice. TCS prevented or inhibited the process of lung tumorigenesis [54]. In another in vivo experiment, TCS elicits an anti-tumor immune response in a murine Lewis lung cancer model by boosting the interaction between tumor suppressor in lung cancer 1 (TSLC1) and class-I MHC-restricted T cell-associated molecule (CRTAM) [55]. TCS inhibits the proliferation of MDA-MB-231 and MCF-7 cells and the growth of transplanted breast cancer in nude mice [56]. For prostatic cancer, trichosanthin can induce apoptosis in RM-1 cells, and the induction of apoptosis is a very important mechanism of trichosanthin to inhibit this type of cancer [57]. TCS can also markedly inhibit melanoma cells by the suppression of DNA synthesis in S phase and cell mitosis as well as induction of cell apoptosis [58].

## Cellular mechanism of TCS

### Cell entry mechanism of TCS

As a toxic protein, TCS needs to enter cells to inactivate the eukaryotic ribosome through its RNA N-glycosidase activity. The first step of this process is that TCS combines and interacts with phospholipids in the cell membrane, because TCS must get across the cell membrane before it can enter the cytoplasm and exert its RIP function [25]. Xia and colleagues investigated the difference between spontaneous and phospholipids-induced adsorption of TCS at the air–water interface. The results were analyzed according to the diffusion–penetration–rearrangement adsorption model. TCS was specific for the negatively charged 1,2-dipalmitoyl-sn-glycero-3-phosphoglycerol (DPPG). Another experiment showed that electrostatic forces dominate the interaction between TCS and negatively charged phospholipid containing membranes under acid conditions. In addition, both the pH value and the ionic strength can influence the binding of TCS molecules [17, 59]. Therefore, electrostatic forces and hydrophobic interaction are proposed to be involved in the binding process of TCS. Xia et al. [60] further investigated the interaction between TCS and a phospholipid bilayer and found that the C-terminus of TCS plays an important role in the interaction between TCS and the membrane.

On the other hand, receptors are also regarded to be necessary for TCS to enter cells [22–24]. TCS enters trophoblasts, JAR cells, and choriocarcinoma BeWo cells by binding to lipoprotein receptor-related protein, LRP1 [26]. It enters proximal tubule epithelial cells by binding to megalin. It inserts into HIV via chemokine receptors [61, 62].

### Inhibition of tumor cell proliferation

TCS suppresses adenylyl cyclase activity and thus reduces cyclic AMP (cAMP) levels in HeLa cells [36]. Interestingly, a decrease in protein kinase C (PKC) level rather than protein kinase A (PKA) level in these cells was observed. This is different from the conventionally accepted mechanism. In another experiment, Wang and colleagues reported that both PKA and PKC activities were significantly inhibited in TCS-treated HeLa cells, although a specific PKA inhibitor failed to affect the effect of TCS, and PKC activator/inhibitor significantly attenuated/enhanced the inhibitory effect of TCS on cell proliferation [33]. The inhibition of PKC was also found to be involved in the apoptotic pathway induced by TCS in K562 cells [45].

The transcriptional factor cAMP response element-binding (CREB) protein, a downstream molecule in cAMP/PKA pathway, was found to participate in the TCS-induced cell death pathway in HeLa cells [35]. CREB phosphorylation was significantly decreased by a cAMP inhibitor, but not by a PKA inhibitor. All these data suggested that HeLa cell proliferation was inhibited by TCS via suppression of the PKC/MAPK signaling pathway.

Furthermore, TCS induced a rapid decline in nuclear factor kappa B (NF-kB) and cyclooxygenase-2 (COX-2) expression leading to apoptosis in hepatoma HepG2 cells [12, 54]. The suppression of NF-kB and of COX-2 protein has been suggested to be important for the antiproliferative and proapoptotic effects on cancer cells [63, 64]. COX-2 may lie downstream of NF-kB, since the inhibition of NF-kB can ensue in down-regulation of COX-2. TCS also down-regulated p210 (Bcr-Abl), protein tyrosine kinase (PTK), and heat shock protein 90 (Hsp90) in chronic myelogenous leukemia K562 cells [46]. All these genes and proteins are associated with proliferation of cancer cells, and their inhibitors have been studied to treat a variety of cancers in clinics [65–68].

**Fig. 1:**
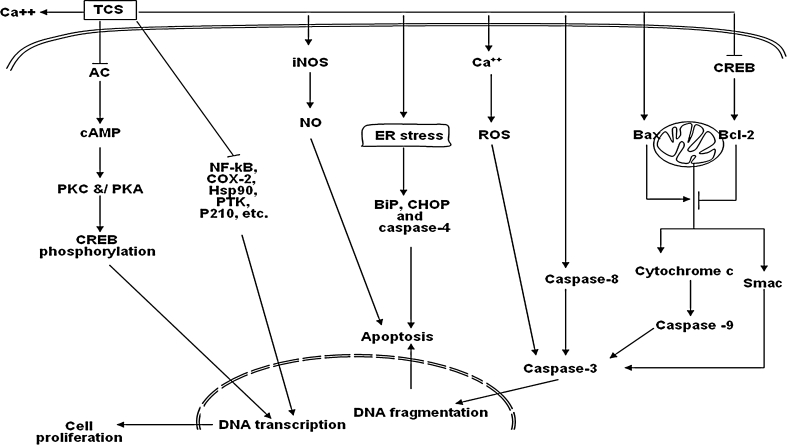
The mechanism of TCS inhibiting proliferation and inducing apoptosis in tumor cells

All abovementioned data infer that TCS inhibits cell proliferation in different tumor cells through different pathways and mechanisms, which merit further in-depth investigations. The mechanisms of TCS are summarized in Fig. 1.

### Apoptosis induced by TCS in tumor cells

#### Increase in Ca^++^ions and reactive oxygen species (ROS)

TCS treatment induced a transient elevation of intracellular calcium and a slow rise of ROS in human chronic myeloid leukemia cell line K562 [45]. However, calcium chelators and antioxidants did not have a conspicuous effect on TCS-induced apoptosis, suggesting that calcium changes and ROS may not be implicated in TCS-mediated apoptosis in K562 cells. In contrast, TCS elicited an increase in cytosolic calcium and induced apoptosis in HeLa cells [36]. Wang et al. [33] also confirmed that this apoptotic cell death can be reduced by a specific calcium chelator, ethylene glycol bis (2-aminoethyl) tetra (acetoxymethyl Ester) (EGTA-AM). The aforementioned discrepancies may be ascribed to the different types of cells studied. TCS may induce apoptosis via distinctly different mechanisms in different cells.

In another cell line, JAR, TCS stimulated the production of ROS, which can be inhibited by the superoxide radical anion (O_2_^−^) scavenger superoxide dismutase, the H_2_O_2_ scavenger catalase, and the hydroxyl radical (OH^−^) scavenger mannitol [28]. The antioxidant Trolox and an inhibitor of metal-facilitated OH^−^ formation, diethylenetriaminepentaacetic acid, also markedly inhibited TCS-induced cell death [27]. All these results indicate that O_2_^−^, H_2_O_2,_ and OH^−^ are involved in TCS-induced ROS formation in JAR cells. The increase in ROS is dependent on the presence of both extracellular and intracellular Ca^++^ ions, and TCS-induced ROS production may be a consequence of Ca^++^ signaling. In addition, TCS-induced activation of caspase 3 was initiated within 2 h; however, TCS-induced production of ROS was initiated within 5 min. These findings suggest that the production of ROS precedes the activation of caspase 3. The apoptotic morphological changes of nuclei were also observed by two-photon laser scanning microscopy in this experiment. The finding that ROS is involved in the TCS-induced apoptosis of JAR cells might provide new insight into the anti-tumor and anti-HIV mechanism of TCS [27, 28].

#### Intrinsic and extrinsic apoptotic pathways

Key caspases in both intrinsic and extrinsic pathways, encompassing caspases 8, 9, and 3, were activated in HeLa-60 cells upon TCS treatment [38]. However, the Fas/Fas ligand pathway was not involved as evidenced by a lack of induction of Fas or Fas ligand and a lack of inhibitory effect of anti-Fas antibody on TCS-induced apoptosis. The involvement of mitochondria was demonstrated by the reduction in mitochondrial membrane potential and release of cytochrome c and Smac besides the activation of caspase 9 [38]. In addition, caspase 3 was corroborated to be the major executioner caspase downstream to caspase 9, 4, and 8. Down-regulation of Bcl-2 was noted in TCS-treated HeLa cells, and CREB is a possible upstream regulator of Bcl-2 in TCS-induced cell apoptosis [33, 35]. On the other hand, protein expression of Bax was up-regulated in TCS-induced apoptosis of murine prostatic cancer RM-1 cells [57].

#### Endoplasmic (ER) pathway and other mechanisms

TCS administration induced upregulation of the protein chaperone BiP and transcription factor CHOP and also activated caspase 4 in HeLa-60 cells, which for the first time strongly supported the involvement of ER stress pathway in TCS-induced apoptosis [38]. On the other hand, inducible nitric oxide synthase (iNOS) mRNA expression and protein levels were elevated in cells treated with TCS, and nitric oxide (NO) production by cells was augmented in the presence of TCS [65]. When L-NIL, the specific inhibitor of iNOS, was added to suppress NO production induced by TCS, OVA-specific cell death was significantly inhibited; meanwhile, cellular thymidine incorporation was restored to normal levels. These observations signify that TCS could suppress antigen-specific T cell activation via an NO-mediated apoptosis pathway. Additionally, TCS can induce specific changes of cytoskeleton configuration associated with the attenuated expression level of actin and tubulin genes in apoptotic HeLa cells [69].

## Conclusion

TCS, a midterm abortion medicine, is a type I RIP endowed with a multiplicity of biological activities, including anti-HIV and anti-tumor functions. TCS has been found to be active against a variety of tumors, including cervical cancer, choriocarcinoma, leukemia/lymphoma, stomach cancer, colon cancer, hepatoma, breast cancer, and prostate cancer. The toxic mechanisms of TCS on tumor cells include inhibition of the proliferation and induction of apoptosis of tumor cells, and the detailed mechanism varies in different tumor cells. Further research on the anti-tumor activities of TCS may not only shed light on cancer therapy, but also on new pharmacological properties of ancient Chinese medicines.
